# Traceability Research on Geographic *Erigeron breviscapus* Based on High-Resolution Mass Spectrometry and Chemometric Analysis

**DOI:** 10.3390/molecules29122930

**Published:** 2024-06-20

**Authors:** Jiao Zhang, Heng Tian, Tao Lin, Xiangzhong Huang, Hongcheng Liu

**Affiliations:** 1 Institute of Quality Standards and Testing Technology, Yunnan Academy of Agricultural Sciences, Agricultural Product Quality Supervision and Inspection Center, Ministry of Agriculture, Kunming 650223, China; cmliu_0@sina.com (J.Z.); tianh@163.com (H.T.); lintaonj@126.com (T.L.); 2 Key Laboratory of Ethnomedicinal Resource Chemistry, Yunnan University for Nationalities, Kunming 650500, China; huangxz@163.com; 3 The Yunnan Provincial Key Lab of Wood Adhesives and Glued Products, Southwest Forestry University, Kunming 650224, China; 4 International Joint Research Center for Biomass Materials, Southwest Forestry University, Kunming 650224, China

**Keywords:** *Erigeron breviscapus*, geographic origin, chemometric analysis

## Abstract

A method was developed to identify and trace the geographic sources of *Erigeron breviscapus* using high-resolution mass spectrometry and chemometrics. The representative samples were collected from the geographic area of Honghe Dengzhanhua and other areas in Yunnan province and Guizhou province. The data points could be determined well using the PCA and PLS-DA diagram. A total of 46 characteristic compounds were identified from Honghe Dengzhanhua and within Guizhou province, but 37 compounds were different from Honghe Dengzhanhua and other counties in Yunnan province. Two biomarkers were found from three regions. Their structures were inferred as 8-amino-7-oxononanoic acid and 8-hydroxyquinoline, and they had the same molecular composition. This may suggest that a possible synthesis pathway can be proven in the future.

## 1. Introduction

Traditional medicines are cultivated in specific regions with a long history of plants. These traditional medicines in a characteristic region are called “orthodox” medicines. *Erigeron breviscapus* is an important traditional medicine. As one of the so-called “Yunyao” varieties, it has been listed as one of the “top ten Yunyao” and “five natural series” medicines in Yunnan province, China [1]. It is known that the best-quality *Erigeron breviscapus* is planted in the Honghe area of Yunnan province, and due to its area of production, is named “Honghe Dengzhanhua” [2]. Although this medicine is identified by its appearance, shape, and main chemical component (scutellarin) in the Chinese national standard, it is difficult to identify the geographic origin of *Erigeron breviscapus* in different plant regions. Since there are tens of major components, Li et al. [3] reported the structure of 64 volatile organic compounds from *Erigeron breviscapus*. However, no biomarker has been found to indicate its geographic origins, so it is urgent to develop an effective traceability technology to identify the geographic production of Honghe Dengzhanhua.

The technology used to determine geographic origin consists of stable-isotope mass spectrometry [4,5], mineral element analysis [6,7], and chromatographic fingerprinting [8,9,10]. However, when an analyte consists of unknown compounds, it is difficult to qualitatively analyze. This problem can be solved by using high-resolution mass spectrometry with nontarget screening technologies [11,12].

Chromatographic fingerprinting involves such complex, multivariate data that it is difficult to distinguish between very similar chromatograms [13]. Thus, the chemical pattern needs to be recognized by using chemometric analysis, such as principal component analysis (PCA) and partial least squares regression (PLS). Xiao et al. (2019) [14] report the classification of *Erigeron breviscapus* from different origins and its related species by HPLC coupled with hierarchical clustering analysis (HCA) and PCA. The multiple linear regression (MLR) and PLS methods were successful in predicting the relationship between compound structure and chromatographic retention time in *Erigeron breviscapus* [3]. The different pattern recognition procedures, including HCA, PCA, and SIMCA, were successful in classifying *G. lucidum* samples through HPLC fingerprinting [15,16]. However, this approach was not designed to trace the geographic production of Honghe Dengzhanhua or discriminate its biomarker compounds in other areas.

In the present study, nontarget screening analysis was carried out alongside high-resolution mass spectrometry, combined with chemometrics, to differentiate the geographic production of Honghe Dengzhanhua from other areas of Yunnan province and Guizhou province, China. In addition, the structures of the characteristic components were elucidated through mass spectrometry. This further proves the possible synthesis of these biomarkers in relation to geographic resources and their bioactivity in the future.

## 2. Materials and Methods

### 2.1. Chemicals and Reagents

Acetonitrile and methanol (HPLC-grade) were supplied by Merck (Darmstadt, Germany). Ammonium acetate and formic acid (HPLC-grade) were purchased from DiKMA Technologies (Beijing, China). Ultrapure water was prepared using Elga’s water system (Wycombe, UK).

### 2.2. Sample Collection and Preparation

**Figure 1:**
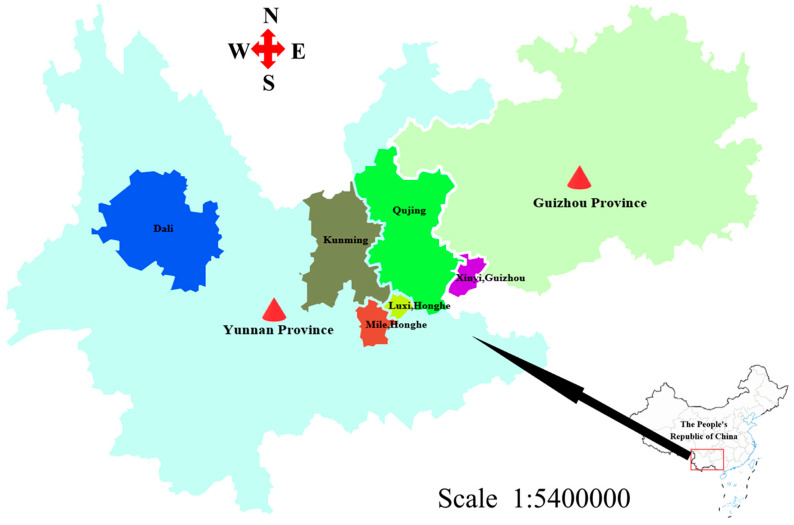
The collected samples from the geographic region of Luxi and Mile, Honghe, and other areas of Yunnan province (Dali, Fuming, Qujing), Xingyi, and Guizhou province.

Thirty samples of *Erigeron breviscapus* were collected from five different areas of Yunnan province and the Xingyi area of Guizhou province, China, in 2022–2023 (Figure 1). For the samples collected in Xingyi City, Guizhou province, the environmental conditions were yellow soil at 16 °C, with a pH of 4.5–6.5, and an altitude of 1200 m; the environmental conditions in Qujing were sandy loam at 18 °C, with a pH of 4.5–7.5, and an altitude of 2000 m. In Honghe, the environmental conditions were red loam and sandy loam at 21 °C with a pH of 6.0 and an altitude of 2459 m. In Dali, the environmental conditions were sandy loam at 17 °C, with a pH of 6–7, and an altitude of 2090 m. In Kunming, the environmental conditions were sandy loam at 15.8 °C, with a pH of 4.0–7.5, and an altitude of 1892 m. The geographical location of the sample is shown in Figure 1. Samples were cut into 4~5 cm lengths, ground into powder at a high speed, passed through a 0.28 µm metal sieve, and stored at 4 °C in the refrigerator.

### 2.3. Sample Preparation and Instrumental Method

Preparation of the Erigeron breviscapus sample: A sample of 1.0 g was precisely weighed and placed in a 50 mL centrifuge tube. After adding 25 mL of a 70% methanol solution, the sample was weighed again. After weighing, the samples were placed in an ice bath at 80 kHz and 25 °C and exposed to ultrasound for 40 min. After the sample was cooled to room temperature, the weight was replenished with a 70% methanol solution. After centrifugation at 5000 r/min for 5 min, the upper liquid was filtered by a 0.22 μm filter membrane, and a high-resolution mass analysis was performed. All of the Erigeron breviscapus samples were mixed equally to obtain QC samples. Then, the QC samples were prepared according to the preparation method used for the Erigeron breviscapus samples (*n* = 6). The preparation of blank samples only required placing 25 mL of 70% methanol in a 50 mL centrifuge tube, according to the method used for the Erigeron breviscapus samples. When the high-resolution injection was used again, the 6-pin blank sample was continuously injected first, and then the 20-pin QC sample was randomly injected and the breviscapine sample was randomly injected and detected. The injection rule is to add one QC sample to each 20-needle Erigeron breviscapus sample, and ensure that the last needle is a QC sample.

The sample was analyzed by Ultra-Performance Liquid Chromatography, using a Q Exactive High-Resolution Mass Spectrometry System (Thermo Fisher Scientific, Rockford, IL, USA) and an HSS T3 C18 column (2.1 × 100 mm, 1.8 µm, Waters, Milford, MA, USA). Solvent A was 0.1% formic acid, and solvent B was acetonitrile. The flow rate was set at 0.3 mL/min with the following gradients programmed: 98% A (0~1.8 min), 98% A~60% A (1.8~10 min), 60% A~5% A (10~12.0 min), 5% A~98% A (12.0~12.5 min), constantly 98% A (12.5~18.0 min) and with 1 µL injection.

The instrument was tuned in the positive and negative ESI mode of 3.8 kV of spray voltage, 60 V of SLens, 325 °C of capillary temperature, and 350 °C of probe heater temperature. Positive calibration solutions and the FS/DIA mode were used. In FS, the scan range was from *m*/*z* 80 to 1200; mass resolution was 140,000 FWHM; and the AGC target and maximum IT were set at 1.0 × 10^6^ and 100 ms, respectively. For DIA, the relevant parameters were set as follows: mass resolution, 140,000 FWHM; AGC target, 2 × 10^5^; maximum IT, 30 ms; Loop count, 12; MSX count, 1; Isolation window, 50 Da; stepped normalized collision energy (NCE), 20%, 40%, and 60%. The spray voltage in positive and negative modes was set as 3.5 kV and 3.0 kV, respectively. The flow rate of sheath gas and aux gas was 45 and 10 mL/min (in arbitrary units), respectively. The software was used with Trace Finder 4.1 EFS and Compound Discoverer 3.3.

#### Data Processing and Statistical Analysis

A response peak area of no more than 400 was ignored, and the ion pairs with a high response intensity were retained. Unsupervised methods (PCA) constituted a first step in the data where samples were clustered with the same compounds. The next steps were to classify by orthogonal partial least squares (OPLS)-DA, and identify more robust samples. The last step was Variable Information Processing analysis (VIP), including a volcano plot (VP) and heat map, in which compounds with a *p*-value < 0.01 and VIP > 1 (top 50) were selected as biomarkers.

The experimental data were analyzed by Compound Discoverer 3.3 software, including peak extraction, peak alignment, background subtraction, compound identification, and multivariate statistical analysis. The peaks extracted by the software were compared with the molecular ion peaks of MzCloud, MzVault, MassLists, and ChemSpider databases in the software through the information of primary and secondary mass spectrometry, and the structural identification of unknown metabolites were realized. Finally, the metabolites with a relative standard deviation of QC sample peak area less than 30% were retained for deduplication. First, internal deduplication was performed according to the priority order in the database: MzVault > MzCloud > MassLists > ChemSpider. When the level was the same, the higher score was selected as the identification result. For the deduplication of positive and negative ion modes, the higher-level results were preferentially retained. If the results were the same, the mode with the largest expression was selected as the reference standard.

## 3. Results

### 3.1. Stability and Repeatability of Instrument

**Figure 2:**
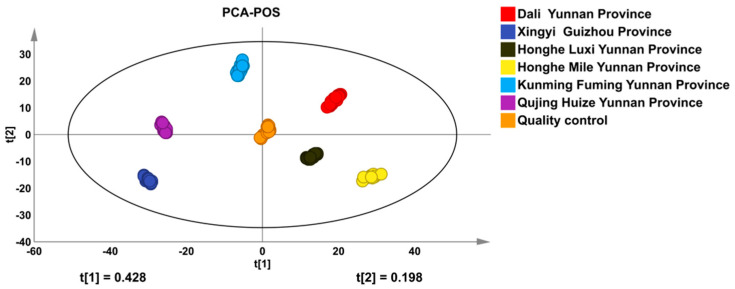

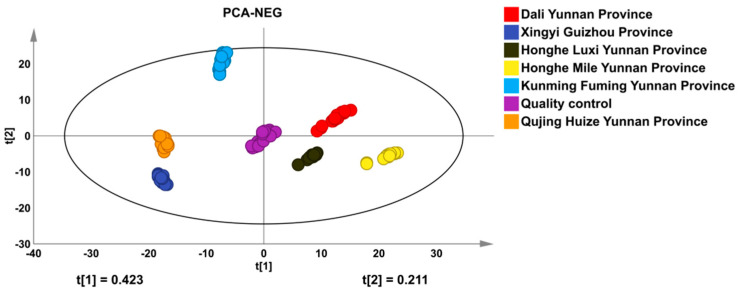
Loading plots of PCA POA (post ion mode) and NEG (negative ion mode) of Honghe Dengzhanhua (I) and other areas. QC: (QC samples).

The quality control samples (QC) were mixed with a total of 120 samples. The sensitivity and stability of the instruments were monitored by QC and corrected for the subsequent data analysis. Each biological replicate of the sample was clustered together and clearly separated from the quality control (QC) sample, indicating that the variation between the samples in the group was the smallest, thus ensuring the repeatability and reliability of the laboratory, as shown in Figure 2. In addition, after consulting the *Pharmacopoeia of the People’s Republic of China*, the main marker compound in the *Erigeron breviscapus* was breviscapine. The RSD is 5.00~6.00%. Through the joint monitoring results for the breviscapine and QC samples, it can be seen that the performance of the instrument is very stable, the experimental repeatability is good, and the obtained data are acceptable and reliable.

### 3.2. Unsupervised Classification by Principal Component Analysis (PCA)

In this analysis, the compounds detected in the sample and the chromatographic peaks corresponding to RSD ≤ 30% in the QC sample were considered to be a variable, and their integrated relative area was the response variable. The compounds selected in this way can be identified as key compounds in *Erigeron breviscapus*, which indicates that the subsequent statistical analysis of the data is credible. The obtained data set from the chemical components retained the maximum possible variability within the conditions. In the study, thirty samples from six areas divided by three classes were drawn in the PCA. The score plot of the PCA (Figure 2) showed that the clear difference in the comparisons was mainly reflected in the difference in the peak areas of the common components from the Honghe region and other areas.

From the scatter plot, the sample could be classified into four groups, including Honghe region, Kunming of Yunnan province, Dali of Yunnan province, and Xingyi of Guizhou province (Figure 2). It can be seen that the Honghe regions of Luxi and Mile can be separated from other areas, whether in Yunnan province or in Guizhou province, but the data were disparate from other areas of Yunnan province. The results show that the chemical composition relates to the plant environment and the climatic character creating biological diversity in Yunnan province. However, the clusters were not obviously distinct between Xingyi county in Guizhou province and Qujing county in Yunnan province. Because the two areas were neighboring, the environmental conditions were similar and associated with the same chemical properties/components. The two principal components, PC1 and PC2, accounted for 44.0% and 19.8% of the total difference, respectively, indicating that these clusters can effectively be distinguished from Honghe Dengzhan and other areas by the differences in the chemical components.

### 3.3. Orthogonal Partial Least Squares Discriminant Analysis (OPLS-DA)

**Figure 3:**
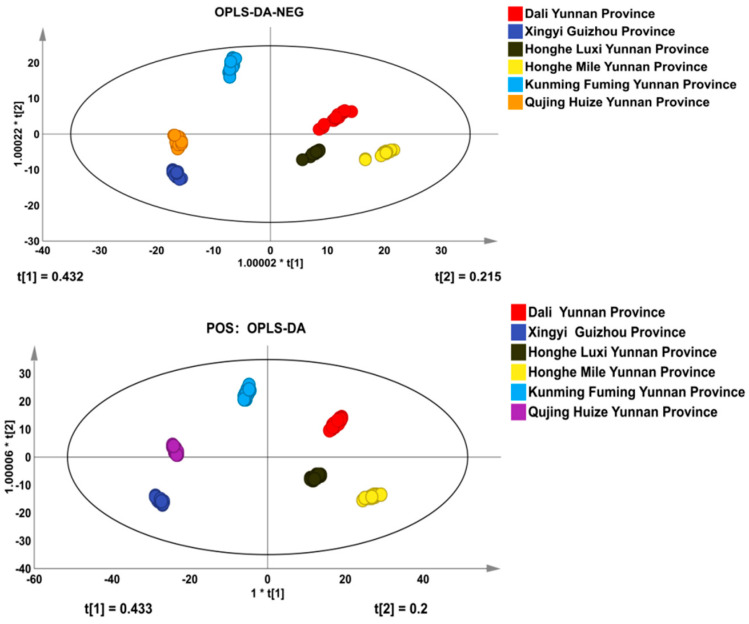
OPLS-DA score chart and model diagram of Honghe and the other four areas.

An appropriate model was established to analyze the collected data, and the OPLS-DA model was obtained. The OPLS-DA was performed to confirm the above four-classes data model. It can discriminate functions with linear combinations of the selected descriptors, and was proportional to the between-classes sum of squares and the within-classes sum of squares. The results were consistent with the PCA, which can be distinguished by the difference in the chemical components from Honghe Dengzhanhua and other areas. The prediction and discrimination ability of the model is greater than 99% (Figure 3).

**Figure 4:**
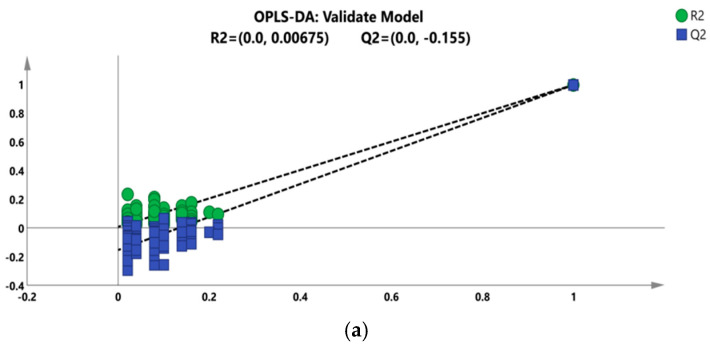

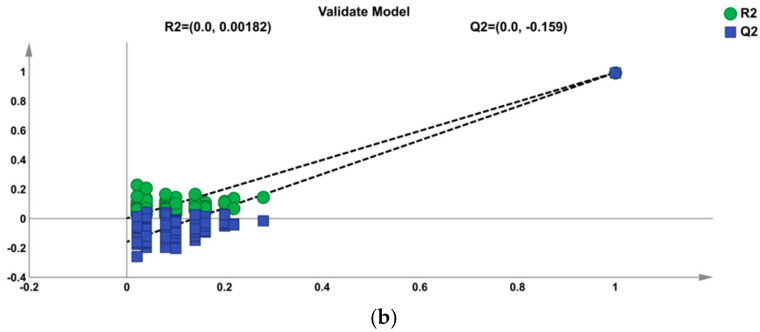
OPLS-DA cross validation details of five areas: (**a**) positive ion mass and (**b**) negative ion mass.

Because OPLS-DA is a supervised discriminant statistical analysis method, a permutation test is used to test the model (Figure 4). In order to avoid over-fitting in the process of model establishment, the intercept of R2 in the permutation test is generally not greater than 0.3~0.4. When the intercept between the Q2 regression line and Y axis (Q2 intercept) is <0.05, it shows that there is no over-fitting phenomenon in the model. The prediction abilities were 100% for all classes. An R2 of 0.99 is the correlation coefficient of cross validation, which indicates that the fitting degree was good. A Q2 of 0.985 represents the performance of the OPLS-DA model that can successfully predict the geographic origin in this experiment (Figure 4).

**Figure 5:**
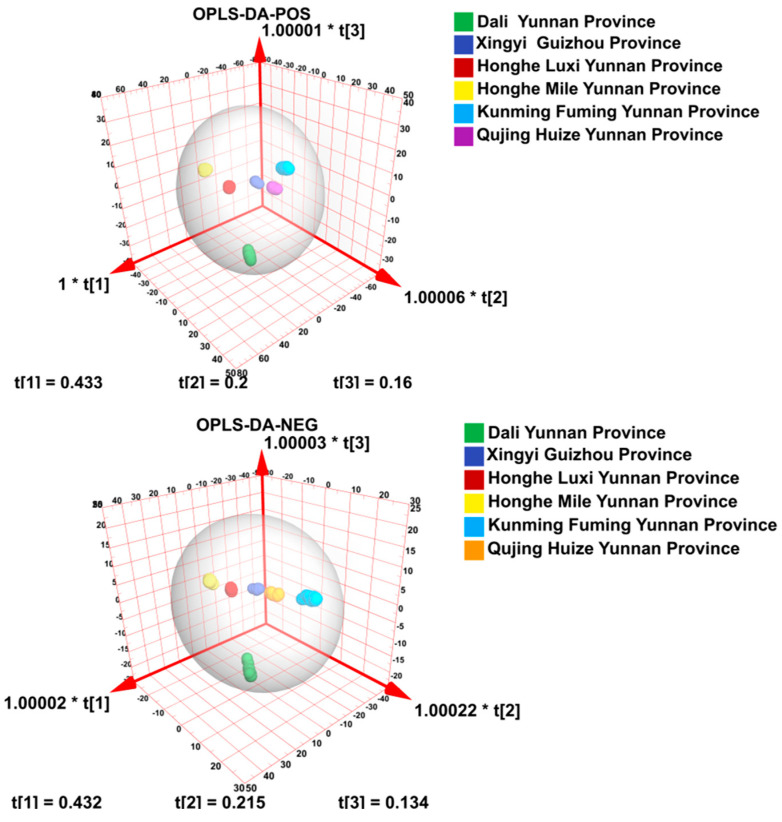
The PLS-DA 3D score plot for the Honghe geographic region and other areas in Yunnan province and Guizhou province.

A 3D score plot was generated to visualize the results from applying OPLS-LDA, as seen in Figure 5. The plot shows the distribution of the samples in the 3D scatter plot. Each point represents a particular class of sample, and is automatically color-coded according to its geographic origin. As can be observed, a complete separation of the four considered classes was achieved (Figure 5).

### 3.4. Variable Information Processing Analysis (VIP), Volcano Plot (VP), and Heat Map

In positive and negative ion toll scan modes, 4852 and 3178 peaks were detected, respectively. The collected mass data were searched using MarkerView 1.3 software (https://cloud.metware.cn). Differential marker compounds were analyzed by *t*-test, when *p* < 0.01 and the fold change (FC) was ≥2 or ≤0.5. The Variable Information Processing analysis (VIP), volcano plot (VP), and heat map were obtained in both positive ion mode and negative ion mode, and the ion pairs with a *p*-value < 0.01 and VIP > 1 were selected to draw plots.

The VIP scores, volcano map, and heat map (see Supplementary Materials, Figures S1–S3) showed the 46 compounds with large differences from Honghe Dengzhanhua and Guizhou provinces. As shown in Table 1, the content of 31 characteristic compounds was larger for Honghe Dengzhanhua than that of other areas, and the content of 15 characteristic compounds of Honghe Dengzhanhua was smaller than that of other areas. The 37 compounds that were differentiated from Honghe Dengzhanhua with other areas in Yunnan province are shown in Figures S1–S3, and the information for these compounds is provided in Table 2. There were 16 characteristic compounds, and their content in Honghe Dengzhanhua was larger than in other areas in Yunnan province, and the content of 21 characteristic compounds in Honghe Dengzhanhua was smaller than the content from other areas in Yunnan province.

### 3.5. Structural Identification of Characteristic Compounds

As an important herb in China, *Erigeron breviscapus* can treat cardiovascular and cerebral vessel diseases with the presence of a number of flavonoids and caffeic acid [55]. Yang reported that caffeic acid was the main constituent in Dengzhan xixin injections [33]. The characteristic components of these compounds may be different for Honghe Dengzhanhua, which mainly includes amino acid, caffeic acid, polyphenols, alkaloids, flavonoids, and their derivatives, as seen in Figure S4.

#### 3.5.1. The Caffeic Acid of Compounds

Caffeic acid and its esters (CAEs) were widely distributed in the herbs as a class of biological activities, including anti-inflammatory, antiviral, anti-atherosclerotic, vasorelaxant, antioxidant, radical-scavenging, immunomodulatory, and hepatoprotective activities [55]. Liao et al. reported [33] that di-*O*-caffeoylquinic acids had characteristic ions at *m*/*z* 353 in *Erigeron breviscapus*. Through a characteristic ion checker of *m*/*z* 353.0876, three peaks were found in Honghe Dengzhanhua (see Supplementary Materials Figure S4). The peak of 6.33 min gave molecular ions at *m*/*z* 353.0874 [M − H]^−^ and characteristic product ions at *m*/*z* 191.0553 ([M − caffeoyl (162) − H]^−^) and 179.0341 ([M − quinic acid − H]^−^), which corresponds to the formula of C_16_H_18_O_9_, but the retention time (7.08 min) was longer than for chlorogenic acid. Through the referred paper [16,46], it is identified as neochlorogenic acid. The peak of 10.20 min gave molecular ions at *m*/*z* 677.14917 [M − H]^−^ and characteristic product ions at *m*/*z* 353.08768 ([M − 2×caffeoyl (334) − H]^−^), 191.0553 ([quinic acid − H]^−^), and 179.0341 ([caffeoyl − H]^−^), which is identified as 1,3,5-tri-*O*-caffeoylquinic acid in Figure S4. Based on the fragmentation behavior, as demonstrated by Yang et al. [56], the peak of 11.08 min was identified as 3,4,9-tri-Caffeoyl-2,7-anhydro-3-deoxy-2-octulopyranosonic acid in Figure S4. The fragmentation pattern of its decaffeoyl product ions was at *m*/*z* 543.11249 ([M − H − 162]^−^), *m*/*z* 381.0844 ([M − H − 334]^−^), and 179.0344 ([caffeic acid − H]^−^). However, the reported di-*O*-caffeoylquinic acids [33] were found, and the content of three compounds were all lower in Honghe Dengzhanhua, which suggests that the three biomarkers were not the main biological activity in Honghe Dengzhanhua.

#### 3.5.2. The Flavonoid of Compounds

Li reported [46] that the three flavonoids of scutellarin, scutellarein, and apigenin were the main compounds in *Erigeron breviscapus* and its extract injection. The results showed that only apigenin was found in Honghe Dengzhanhua, and the contents were higher, which suggests that the content of apigenin was high and bioactive in Honghe Dengzhanhua. Furthermore, a new polyphenol was found with the same molecular makeup of scutellarein, but it had a different retention time. The new polyphenols were identified at 7.38 min, which was earlier than for scutellarein at 8.91 min. The new polyphenols belonged to ellagic acid, which gave molecular ions at *m*/*z* 463.0868 [M + H]^+^ and characteristic product ions at *m*/*z* 289.0341 ([M − arabinose-2CH_3_ + H]^+^) and 243.02905 ([quinic acid + H]^+^), corresponding to the formula of C_21_H_18_O_12_ and presumed as 2-hydroxy-3,8-dimethoxy-7-((3,4,5-trihydroxytetrahydro-2*H*-pyran-2-yl)oxy)chromeno[5,4,3-cde]chromene-5,10-dione (Supplementary Materials Figure S4), which shows potent inhibition without cytotoxicity [44].

The other flavonoid was found at the peak of 8.48 min that gave molecular ions at *m*/*z* 677.14917 [M − H]^−^ and characteristic product ions at *m*/*z* 353.08768 ([M − 2×caffeoyl (334) − H]^−^), 191.0553 ([quinic acid − H]^−^), and 179.0341 ([caffeoyl − H]^−^), which corresponded to the formula of C_20_H_18_O_11_, presumed to be guaijaverin, as seen in Supplementary Materials Figure S4. The guaijaverin was shown to exert a dose-dependent inhibition of DP-IV [31]. It was first found in *Erigeron breviscapus*. The peak of 11.55 min gave molecular ions at *m*/*z* 677.14917 [M − H]^−^ and characteristic product ions at *m*/*z* 353.08768 ([M − 2×caffeoyl (334) − H]^−^), 191.0553 ([quinic acid − H]^−^), and 179.0341 ([caffeoyl − H]^−^). It is presumed to be Pongamoside C [57], as seen in Supplementary Materials Figure S4. The peak of 9.06 min gave molecular ions at *m*/*z* 193.04977 [M + H]^+^ and characteristic product ions at *m*/*z* 178.02617 ([M − CH_3_+H]^+^) and 133.02856 ([qM − CH_3_-COOH + H]^−^). It corresponded to the formula of C_10_H_8_O_4_, and was inferred to be Scopoletin [43].

### 3.6. The Simultaneous Biomarkers Distinguished in Honghe Dengzhanhua from Other Areas in Yunnan Province, Guizhou Province

The relative content of two biomarkers from Honghe Dengzhanhua was higher than that from Guizhou province and other areas in Yunnan province, which may be mainly attributed to the geographic resources of Honghe Dengzhanhua. The peak of 6.28 min gave molecular ions at *m*/*z* 188.07068 [M + H]^+^ and characteristic product ions at *m*/*z* 146.0602 ([M − COCH_3_ + H]^+^) and 118.0655 ([M − COCH_3_-CHOH + H]^+^). It corresponded to the formula of C_11_H_9_NO_2_, and was presumed to be 2-Acetyl-quinoline-8-ol, as seen in Supplementary Materials Figure S4. This heterocyclic structure is an important bioactive molecule [58]. In particular, 8-hydroxyquinolines have been widely used in therapeutics, demonstrating an array of medicinal applications (e.g., antibacterial, anti-cancer, anti-neurodegenerative, antiviral) [21]. Except for 2-Acetyl-quinoline-8-ol, other quinolines were found, as presented in Table 1 and Table 2. They were 8-Hydroxyquinoline and 2-Methyl-8-quinolinamine, and all included 118.0655 ([M − COCH_3_-CHOH + H]^+^), which means that these quinoline metabolites will be important bioactive compounds in Honghe Dengzhanhua.

**Figure 6:**
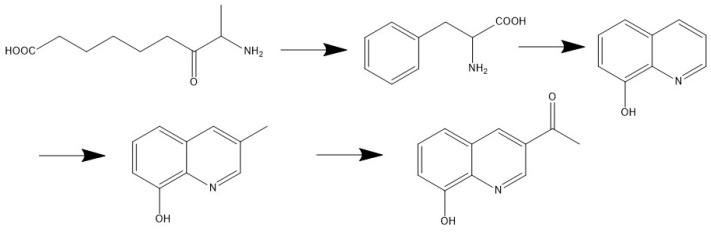
The possible synthesis of the two biomarkers in Honghe Dengzhanhua.

The other peak of 3.00 min gave molecular ions at *m*/*z* 188.12839 [M + H]^+^ and characteristic product ions at *m*/*z* 142.12280 ([M − COOH-H]^+^) and 74.0608 ([M − COOH-C_4_H_8_-H]^+^). It corresponded to the formula of C_9_H_17_NO_3_ and was inferred to be 8-amino-7-oxononanoic acid, as seen in Supplementary Materials Figure S4. Amino ketones are important structural moieties that are widely found in many pharmaceuticals and bioactive compounds. They are also valuable intermediates for the synthesis of complex compounds, including 2-amino alcohols and various *N*-heterocycles [18]. The 8-amino-7-oxononanoic acid and 8-hydroxyquinoline had the same molecular composition, suggesting that a possible synthesis pathway may exist, as seen in Figure 6. It further proves the possible synthesis of the two biomarkers with the relationship to geographic resources and their bioactivity in the future.

## 4. Conclusions

*Erigeron breviscapus* is widely used as a traditional medicine material. Although the chemical and biological morphology is described by the Chinese national standard, it is difficult to distinguish the geographic origin with other areas in Yunnan province and Guizhou province. Thus, chemometrics should be developed to distinguish functions with linear combinations of the selected descriptors of the geographic regions. In this work, a total of 424 metabolites in whole quinoa grains were detected and annotated, which included 35 compounds in Honghe Dengzhan and Guizhou province, and 37 compounds in Honghe Dengzhan and other areas in Yunnan province. The two candidate biomarker constituents were determined by grouping discriminant compounds, which suggests that the possible synthesis of the two candidate biomarkers can be proven in the future. However, the differences in the metabolites produced in different geographic regions and cultivars have not been thoroughly researched. Overall, this research advances our knowledge of the metabolic mechanisms in geographic regions and lays a firm foundation for the further cultivation of orthodox pharmacy.

## Figures and Tables

**Table 1:** Difference biomarker in Honghe Dengzhan and Guizhou province analyzed by High resolution mass spectrum.

Classification	Name	Formula	*m*/*z*	Mass Error [ppm]	RT [min]	Adduct/Charge	Reference	Changes of Content in Honghe Dengzhanhua
amino acid	Phenylalanine	C_9_H_11_NO_2_	166.08627	0.1	4.709	[M + M]^+^	[17]	up
Polyphenols	Ghanamycin A	C_13_H_16_O_9_	317.0866	−0.37	6.479	[M + H]^+^	unknown	up
Polyphenols	Ghanamycin B	C_19_H_28_O_9_	401.1802	−1.13	9.069	[M + H]^+^	unknown	up
amino acid	Dimethyl *N*-[2-hydroxy-4-methoxy-2-(2-methoxy-2-oxoethyl)-4-oxobutanoyl]glutamate	C_15_H_23_NO_10_	360.12882	−0.16	5.693	[M + H]^+^	[18]	up
Polyphenols	Ghanamycin A	C_13_H_16_O_9_	317.08663	−0.27	5.54	[M + H]^+^	Unknown	up
amino acid	(2S,3R,4S,5R,6R)-2-{[(E)-{2-[(2R,5S,6R)-5-Acetoxy-6-(acetoxymethyl)-5,6-dihydro-2*H*-pyran-2-yl]ethylidene}amino]oxy}-6-(acetoxymethyl)tetrahydro-2*H*-pyran-3,4,5-triyl triacetate	C_26_H_35_NO_15_	602.20782	−0.21	4.695	[M + H]^+^	Unknown	up
Polyphenols	3,4,5-Trimethoxyphenyl 2,4-dideoxy-6-*O*-[(2R,3R,4R)-3,4-dihydroxy-4-(hydroxymethyl)tetrahydro-2-furanyl]-beta-d-threo-hexopyranoside	C_20_H_30_O_11_	447.18575	−0.78	9.408	[M + H]^+^	Unknown	up
amino acid	Salbostatin	C_13_H_23_NO_8_	322.14928	−1.14	8.402	[M + H]^+^	[19]	up
amino acid	MFCD00025555	C_9_H_18_N_2_O_3_	203.13901	−0.04	5.33	[M + H]^+^	Unknown	up
amino acid	D-(+)-Tryptophan	C_11_H_12_N_2_O_2_	205.0971	−0.27	6.322	[M + H]^+^	[20]	up
alkaloid	2-Methyl-8-quinolinamine	C_10_H_10_N_2_	159.09163	−0.26	6.322	[M + H]^+^	[21]	up
alkaloid	2-Acetyl-quinoline-8-ol	C_11_H_9_NO_2_	188.07061	0.01	6.322	[M + H]^+^	[21]	up
amino acid	Neuraminic acid	C_9_H_17_NO_8_	268.10394	4.69	2.975	[M + H]^+^	[22]	up
Polyphenols	Ralfuranone A	C_10_H_8_O_2_	193.08593	0.04	7.001	[M + H]^+^	[23]	up
amino acid	4-Pyridine carbohydrazide	C_5_CH_7_N_3_O	317.08664	−1.96	6.481	[M + H]^+^	[24]	up
Polyphenols	O-methyl melleine	C_11_H_12_O_3_	193.08589	−0.45	10.665	[M + H]^+^	[25]	up
alkaloid	(6R)-3,5-Dideoxy-5-{[(3-methyl-2-oxo-4a,8a-dihydro-2*H*-chromen-7-yl)carbonyl]amino}-6-[(1R,2R)-1,2,3-trihydroxypropyl]-alpha-l-threo-hex-2-ulopyranosonic acid	C_20_H_25_NO_11_	456.1498	−0.52	2.199	[M + H]^+^	Unknown	up
amino acid	Panclicin D	C_25_H_45_NO_5_	440.3368	−0.58	4.707	[M + H]^+^	[26]	up
alkaloid	(1R)-1,5-Anhydro-1-({(5S)-3-[(3aS,4R,6R,6aS)-6-hydroxy-2,2-dimethyltetrahydrofuro[3,4-d][1,3]dioxol-4-yl]-4,5-dihydro-1,2-oxazol-5-yl}methyl)-d-galactitol	C_17_H_27_NO_10_	406.17043	−0.84	2.28	[M + H]^+^	unknown	up
amino acid	(2R,3S)-7-Acetamido-6-acetoxy-1,2,3-octanetriyl triacetate	C_18_H_29_NO_9_	436.21755	−0.42	9.312	[M + H]^+^	unknown	up
amino acid	(S)-malyl-d-glucosaminide	C_10_H_17_NO_9_	296.09749	−0.39	1.88	[M + H]^+^	[21]	up
amino acid	4-Hydroxy-3-methoxybenzyl 2-acetamido-2-deoxy-beta-d-glucopyranoside	C_16_H_23_NO_8_	390.17567	−0.53	7.002	[M + H]^+^	Unknown	up
amino acid	8-Amino-7-oxononanoic acid	C_9_H_17_NO_3_	188.12806	−0.3	3.03	[M + H]^+^	[18]	up
amino acid	alpha-L-Rhap-(1->3)-beta-D-GlcpO[CH2]5NH2	C_17_H_33_NO_10_	412.21752	−0.5	8.403	[M + H]^+^	[27]	up
amino acid	2-{[2-{[(6-Aminohexanoyl)oxy]methyl}-2-(hydroxymethyl)butoxy]carbonyl}cyclohexanecarboxylic acid	C_20_H_35_NO_7_	402.24842	−0.58	9.323	[M + H]^+^	Unknown	up
amino acid	*N*-(tert-Butoxycarbonyl)-L-glutamine	C_10_H_18_N_2_O_5_	247.12875	−0.36	4.501	[M + H]^+^	[28]	up
Polyphenols	1-O-[(2E,4Z,7Z)-2,4,7-Decatrienoyl]-2-O-beta-d-glucopyranosyl-beta-d-glucopyranose	C_22_H_34_O_12_	491.21206	−0.8	9.167	[M + H]^+^	[29]	up
acid	5-(Ethoxycarbonyl)-7-oxabicyclo[2.2.1]heptane-2,3-dicarboxylic acid	C_11_H_14_O_7_	259.08103	−0.79	8.402	[M + H]^+^	[30]	up
flavonoids	Guaijaverin	C_20_H_18_O_11_	435.09183	−0.83	8.498	[M + H]^+^	[31]	up
amino acid	Dimethyl N,N-bis{[(2-methyl-2-propanyl)oxy]carbonyl}-L-glutamate	C_17_H_29_NO_8_	376.19588	−1.9	8.849	[M − H]^-^	unknown	up
alkaloid	(S)-5-(4-hydroxybenzoyl)-3-isobutyrylimidazolidine-2,4-dione	C_14_H_14_N_2_O_5_	291.09716	−1.35	9.108	[M + H]^+^	[32]	up
alkaloid	Methyl 4-(4-methyl-1-piperazinyl)-3-nitrobenzoate	C_13_H_17_N_3_O_4_	280.12912	−0.23	5.65	[M + H]^+^	unknown	down
caffeoyl	1,3,5-tri-*O*-caffeoylquinic acid	C_34_H_30_O_15_	679.16532	−0.63	10.208	[M + H]^+^	[33]	down
amino acid	3-hydroxy-2-N-iso-butyryl-anthranilamide	C_11_H_14_N_2_O_3_	223.10758	−0.62	2.436	[M + H]^+^	[34]	down
alkaloid	MFCD00023832	C_12_H_18_N_2_O_4_	237.1233	−0.29	5.963	[M + H]^+^	Unknown	down
amino acid	FW054-1	C_15_H_21_NO_6_	312.14393	−0.74	4.61	[M + H]^+^	Unknown	down
alkaloid	8-Hydroxyquinoline	C_9_H_7_NO	146.05998	−0.39	6.321	[M + H]^+^	[21]	down
alkaloid	2-Hydroxy-5-(3,5,7-trihydroxy-4-oxo-4H-chromen-2-yl) phenyl 6-aminohexanoate	C_21_H_21_NO_8_	416.13367	−0.77	8.675	[M + H]^+^	unknown	down
alkaloid	Isopimara-8,15-dien-19-ol	C_20_H_32_ O	289.25222	−1.21	11.983	[M + H]^+^	[35]	down
amino acid	3-(4-Hydroxy-3-methoxyphenyl)propyl 2-acetamido-2-deoxy-beta-d-glucopyranoside	C_18_H_27_NO_8_	386.18053	−1.07	10.665	[M + H]^+^	[36]	down
amino acid	(2R,3R,4R,5S,2′R,3′R,4′R,5′S)-6,6′-[(2-Phenylethyl)imino]di(1,2,3,4,5-hexanepentol)	C_20_H_35_NO_10_	450.23328	−0.21	9.183	[M + H]^+^	[37]	down
alkaloid	6-Nitro-1,2,3-benzotriazin-4(1H)-one 2-oxide	C_7_H_4_N_4_O_4_	209.0305	−0.16	3.202	[M + H]^+^	[38]	down
Polyphenol	Usimine A	C_24_H_25_NO_10_	488.15493	−0.39	11.568	[M + H]^+^	unknown	down
Polyphenol	(2R,4R)-3,4-dihydro-5-methoxy-2-methyl-2*H*-1-benzopyran-4-ol	C_11_H_14_O_3_	195.1015	−0.41	10.16	[M + H]^+^	[39]	down
Amino acid	Stearoyl glutamic acid	C_23_H_43_NO_5_	414.32123	−0.42	12.421	[M + H]^+^	[40]	down
Flavone	Pongamoside C	C_24_H_22_O_10_	471.12845	−0.37	11.568	[M + H]^+^	[41]	down

**Table 2:** Difference biomarker in Honghe Dengzhan and other area in Yunnan province analyzed by High resolution mass spectrum.

Classification	Name	Formula	*m*/*z*	Mass Error [ppm]	RT [min]	Adduct/Charge	Reference	Changes of Content in Honghe Dengzhanhua
amino acid	Epinephrine glucuronide	C_15_H_21_NO_9_	360.12881	−0.23	5.696	[M + M]^+^	[41]	up
alkaloid	(S)-5-(4-hydroxybenzoyl)-3-isobutyrylimidazolidine-2,4-dione	C_14_H_14_N_2_O_5_	291.09716	−1.35	9.108	[M + H]^+^	[32]	up
amino acid	8-Amino-7-oxononanoic acid	C_9_H_17_NO_3_	188.12806	−0.3	3.03	[M + H]^+^	[18]	up
amino acid	*N*-(tert-Butoxycarbonyl)-l-glutamine	C_10_H_18_N_2_O_5_	247.12875	−0.36	4.501	[M + H]+	[28]	up
amino acid	Serpulanine C	C_14_H_18_N_2_O_3_	263.13895	−0.23	6.747	[M + H]+	[42]	up
amino acid	Salbostatin	C_13_H_23_NO_8_	322.14928	−1.14	8.402	[M + H]^+^	[19]	up
alkaloid	2-Acetyl-quinoline-8-ol	C_11_H_9_NO_2_	188.07061	0.01	6.322	[M + H]^+^	[21]	up
alkaloid	Scopoletin	C_10_H_8_O_4_	193.04947	−0.32	9.093	[M + H]^+^	[43]	up
polyphenols	2-hydroxy-3,8-dimethoxy -7-((3,4,5-trihydroxytetrahydro -2*H*-pyran-2-yl)oxy) chromeno [5,4,3-cde]chromene-5,10-dione	C_21_H_18_O_12_	463.08705	−0.12	7.38	[M + H]^+^	[44]	up
Polyphenols	Bis{2-[2-(methacryloyloxy)ethoxy]ethyl} 4-cyclohexene-1,2-dicarboxylate	C_24_H_34_O_10_	483.22221	−0.55	12.224	[M + H]^+^	unknown	up
alkaloid	(1R)-1,5-Anhydro-1-({(5S)-3-[(3aS,4R,6R,6aS)-6-hydroxy-2,2-dimethyltetrahydrofuro[3,4-d][1,3]dioxol-4-yl]-4,5-dihydro-1,2-oxazol-5-yl}methyl)-d-galactitol	C_17_H_27_NO_10_	406.17043	−0.84	2.28	[M + H]^+^	unknown	up
acid	M-hydroxyphenyl acetic acid	C_7_H_6_O_3_	139.03896	0.27	6.766	[M + H]^+^	[21]	up
Polyphenols	4-(beta-d-Glucosyloxy)benzoate	C_13_H_16_O_8_	323.07363	−0.34	6.765	[M + H]^+^	[45]	up
flavonoids	Apigenin	C_15_H_10_O_5_	271.05984	−0.98	12.137	[M + H]^+^	[46]	up
alkaloid	Tabtoxin	C_11_H_19_N_3_O_6_	290.13446	−0.68	1.65	[M + H]^+^	[47]	up
alkaloid	4-Nitrobenzyl 2-oxo-2*H*-chromene-3-carboxylate	C_17_H_11_NO_6_	326.06543	−1.5	9.7	[M + H]^+^	unknown	up
Amino acid	3,4-Dimethoxybenzyl 2-acetamido-2-deoxy-beta-d-glucopyranoside	C_17_H_25_NO_8_	372.16512	−0.47	10.666	[M + H]^+^		[48]
Polyphenols	Pestalotheol G	C_16_H_22_O_6_	311.14853	−1.28	10.976	[M + H]^+^	[49]	down
Polyphenols	O-methyl melleine	C_11_H_12_O_3_	193.08589	−0.45	10.665	[M + H]^+^	[25]	down
Amino acid	Stearoyl glutamic acid	C_23_H_43_NO_5_	414.32123	−0.42	12.421	[M + H]^+^	[40]	down
amino acid	Valilactone	C_22_H_39_NO_5_	398.28979	−0.81	11.671	[M + H]^+^	[50]	down
Polyphenols	Gloeolactone	C_18_H_28_O_3_	293.21063	−1.33	11.302	[M + H]^+^		down
Polyphenols	9-epi-sacrolide A	C_18_H_28_O_4_	309.20569	−0.98	11.619	[M + H]^+^	[51]	down
Polyphenols	Ralfuranone A	C_10_H_8_O_2_	193.08587	−0.36	10.665	[M + H]^+^	[23]	down
acid	Lorneic acid B	C_17_H_24_O_3_	309.20594	−0.8	11.391	[M + H]^+^	[23]	down
amino acid	Curvularide A	C_18_H_35_NO_5_	346.25842	−1.1	11.475	[M + H]^+^	[52]	down
amino acid	Tributyl 2,2′,2″-nitrilotriacetate	C_18_H_33_NO_6_	360.23764	−1.17	11.89	[M + H]^+^	unknown	down
amino acid	3-(4-Hydroxy-3-methoxyphenyl)propyl 2-acetamido-2-deoxy-beta-d-glucopyranoside	C_18_H_27_NO_8_	386.18053	−1.07	10.665	[M + H]^+^	unknown	down
Polyphenols	Tributyl Aconitate	C_18_H_30_O_6_	343.21083	−1.86	11.896	[M + H]^+^	[53]	down
caffeoyl	1,3,5-tri-*O*-caffeoylquinic acid	C_34_H_30_O_15_	679.16575	−1.13	11.02	[M + H]^+^	[33]	down
caffeoyl	3,4,9-tri-Caffeoyl-2,7-anhydro-3-deoxy-2-octulopyranosonic acids	C_35_H_30_O_16_	707.16028	−0.54	11.156	[M + H]^+^	[33]	down
amino acid	Dimethyl 4-acet amidodecanedioate	C_14_H_25_NO_5_	288.18028	−0.83	7.673	[M + H]^+^	[54]	down
amino acid	butoctamide	C_16_H_29_NO_5_	316.21145	−1.27	9.201	[M + H]^+^	unknown	down
caffeoyl	Neochlorogenic acid	C_16_H_18_O_9_	355.10218	−0.86	6.281	[M + H]^+^	[46]	down

## Data Availability

The raw data supporting the conclusions of this article will be made available by the authors on request.

## Supplementary Materials

The following supporting information can be downloaded at: https://www.mdpi.com/article/10.3390/molecules29122930/s1. Detailed information on the 424 metabolites identified in *Erigeron breviscapus* from different areas of two provinces is listed in Tables S1–S3. The VIP scores of samples, a volcano plot, a clustering heat map, and a total ion chromatogram (TIC) mass spectrum is shown in Figures S1–S4.
