# Supplementary material for: Traceability Research on Geographic Erigeron breviscapus Based on High-Resolution Mass Spectrometry and Chemometric Analysis
Source: Molecules. 2024 Jun 20;29(12):2930. doi: 10.3390/molecules29122930 (PMC11206744; doi:10.3390/molecules29122930)
Supplement: Supplementary file 1 [file molecules-29-02930-s001.zip › Figure S4.pdf]

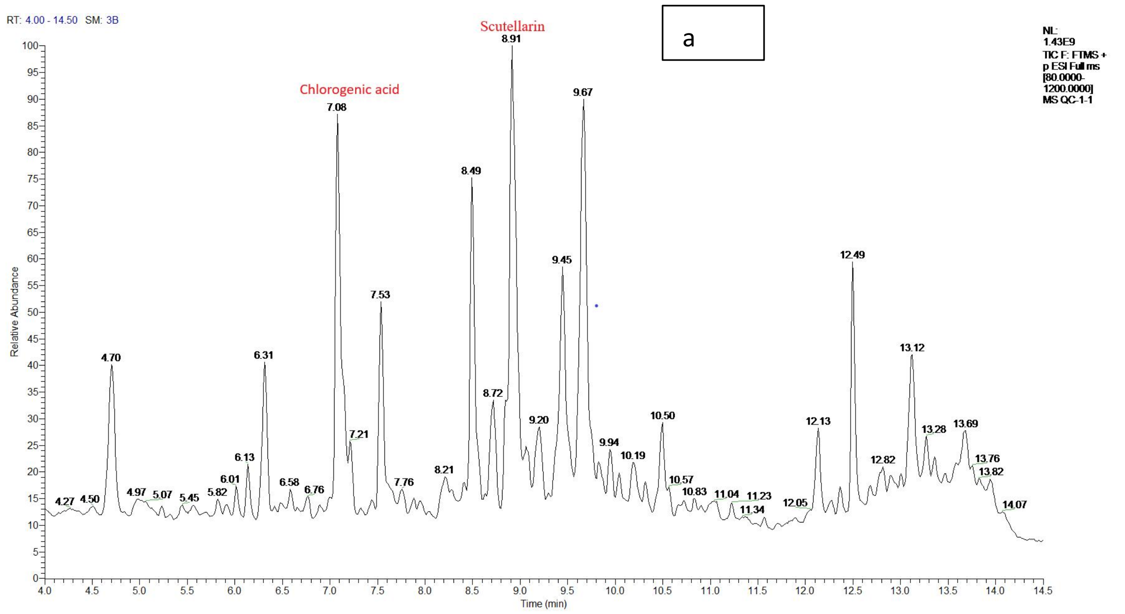

Figure S4 the scan chramatogram

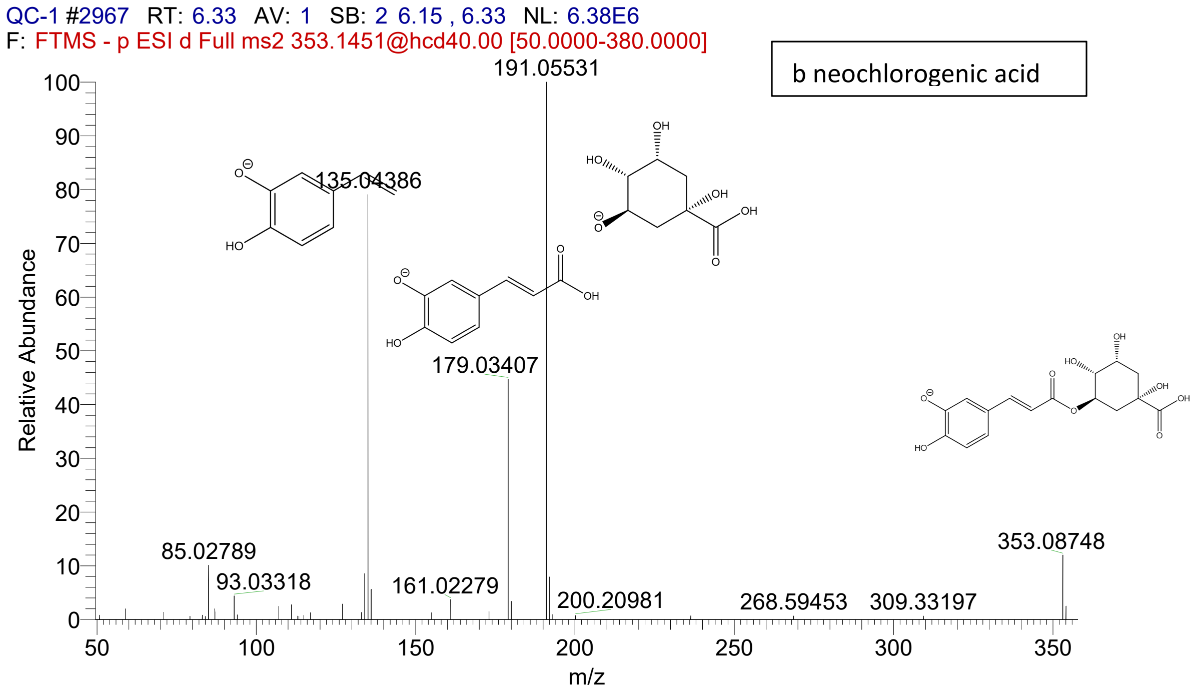

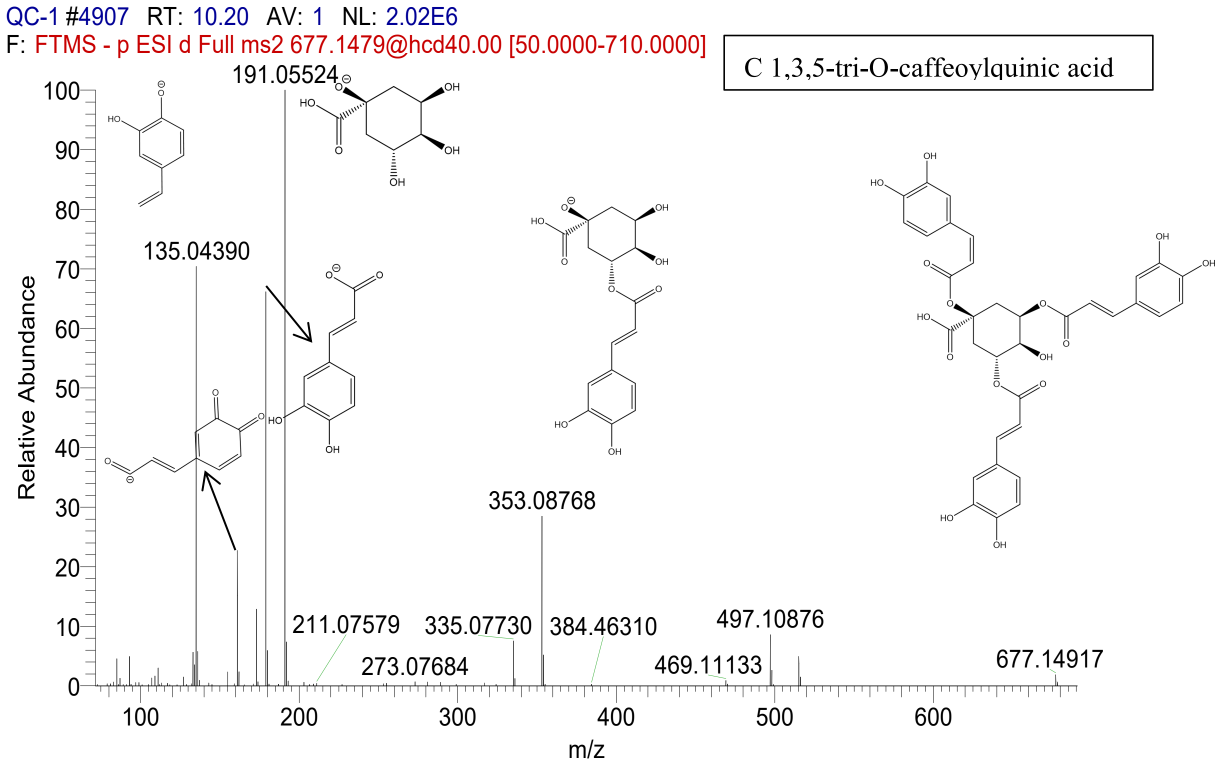
QC-1 #4907 RT: 10.20 AV: 1 NL: 2.02E6
F: FTMS - p ESI d Full ms2 677.1479@hcd40.00 [50.0000-710.0000]
C 1,3,5-tri-O-caffeoylquinic acid
191.05524
135.04390
353.08768
211.07579
335.07730
384.46310
497.10876
273.07684
469.11133
677.14917
Relative Abundance
m/z

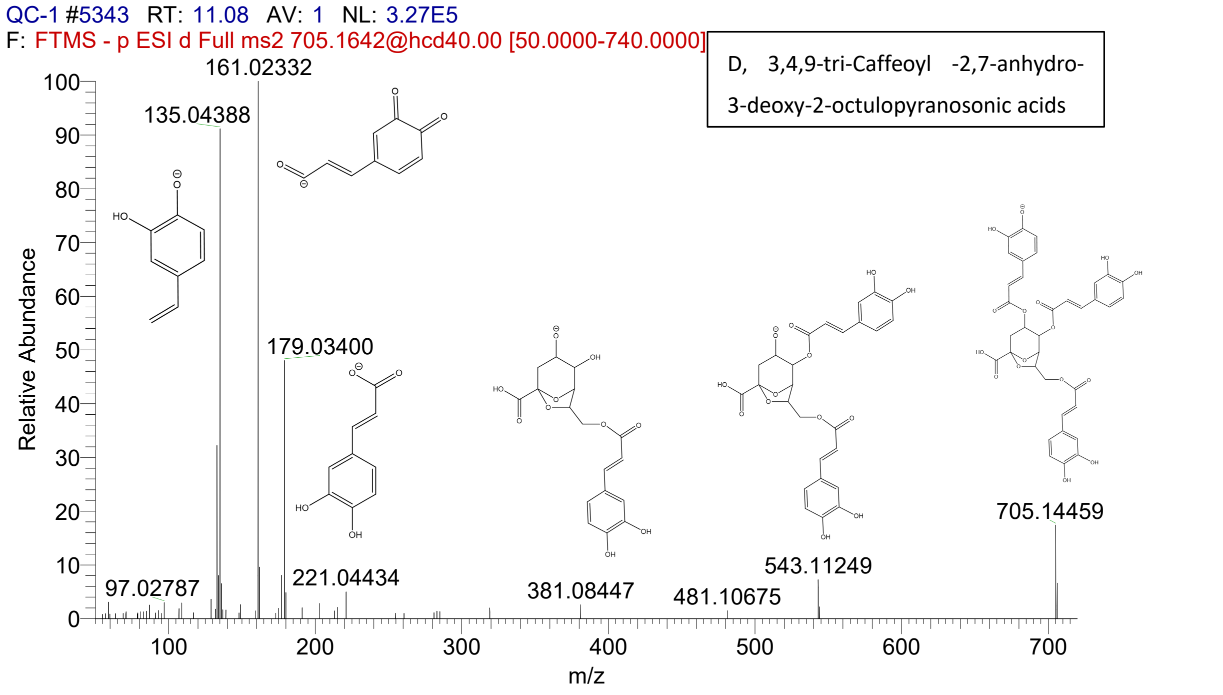
QC-1 #5343 RT: 11.08 AV: 1 NL: 3.27E5
F: FTMS - p ESI d Full ms2 705.1642@hcd40.00 [50.0000-740.0000]
D, 3,4,9-tri-Caffeoyl -2,7-anhydro-3-deoxy-2-octulopyranosonic acids
161.02332
135.04388
179.03400
705.14459
543.11249
97.02787
221.04434
381.08447
481.10675
Relative Abundance
m/z

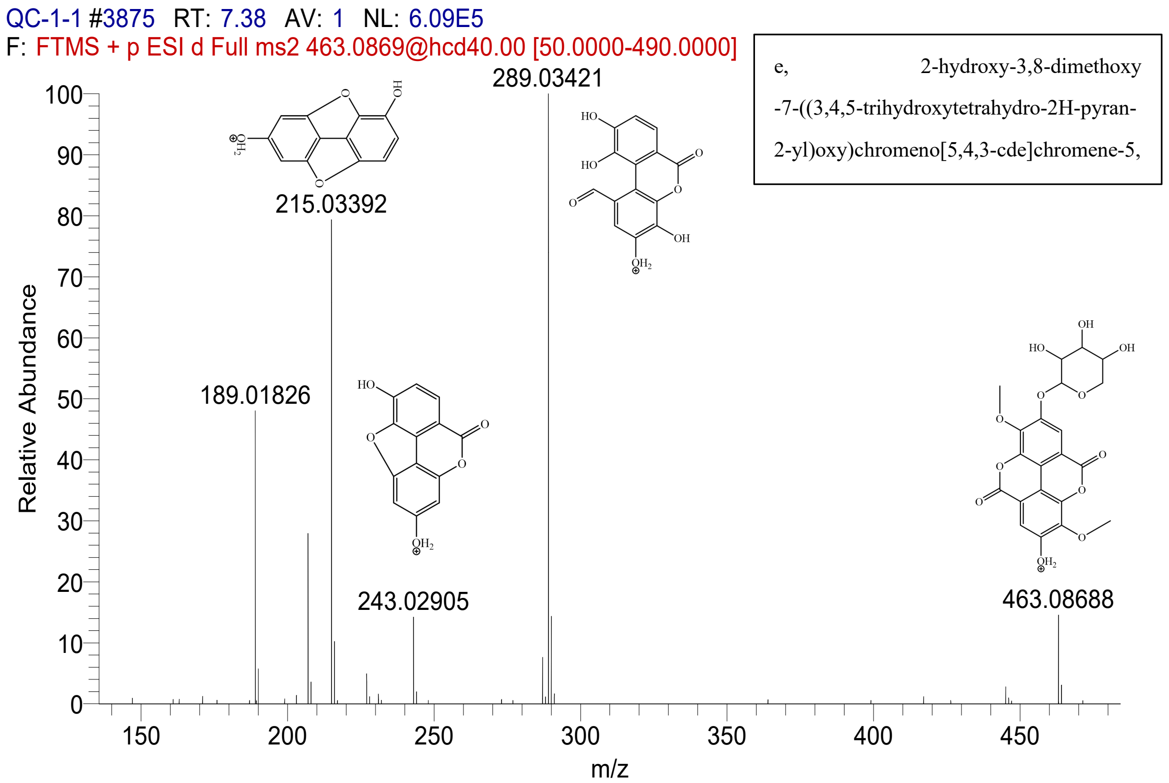

e, 2-hydroxy-3,8-dimethoxy -7-((3,4,5-trihydroxytetrahydro-2H-pyran-2-yl)oxy)chromeno[5,4,3-cde]chromene-5,

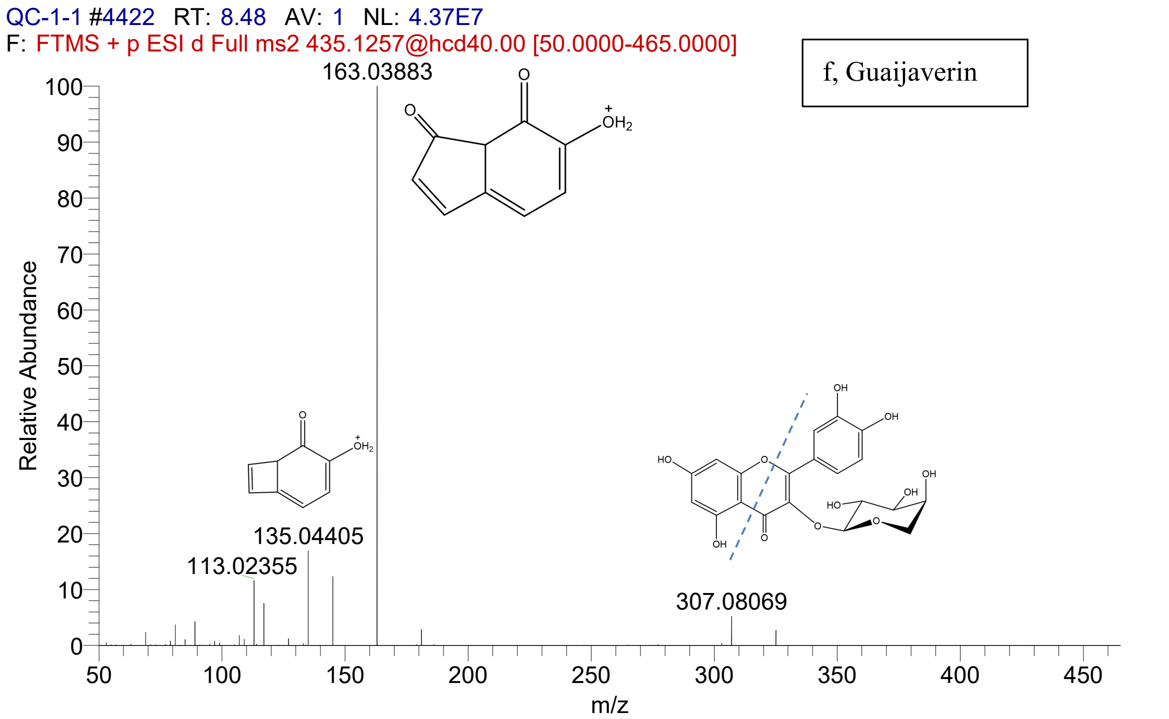

f, Guaijaverin

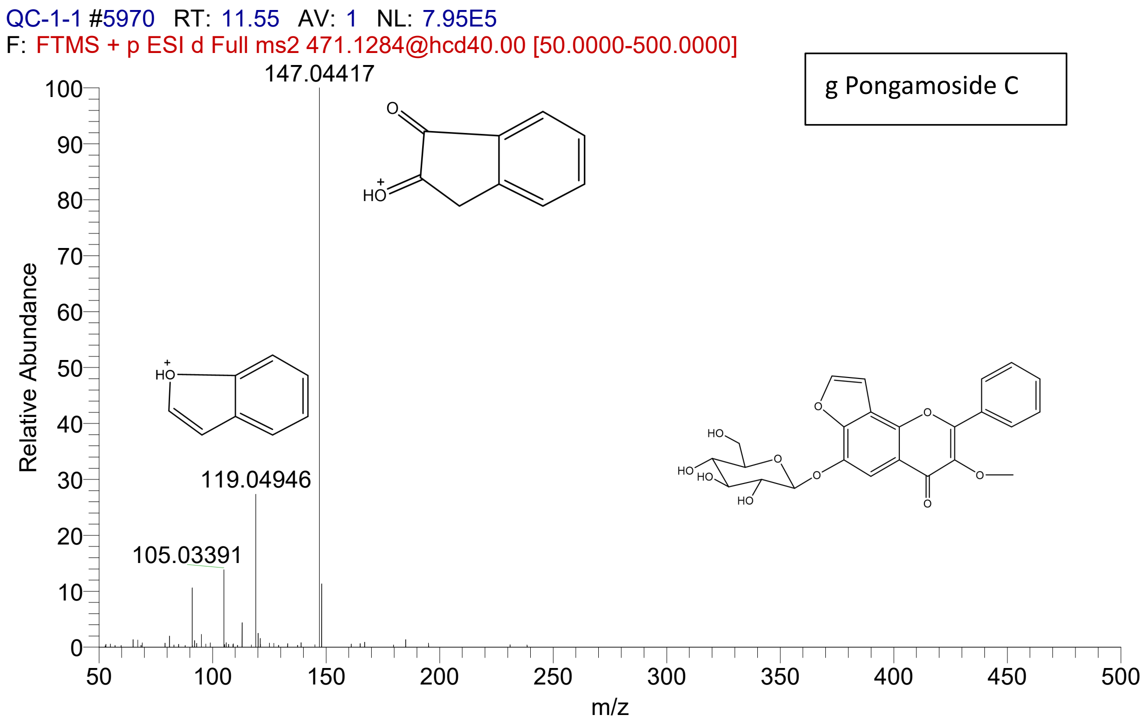
QC-1-1 #5970 RT: 11.55 AV: 1 NL: 7.95E5
F: FTMS + p ESI d Full ms2 471.1284@hcd40.00 [50.0000-500.0000]
g Pongamoside C
147.04417
119.04946
105.03391
Relative Abundance
m/z

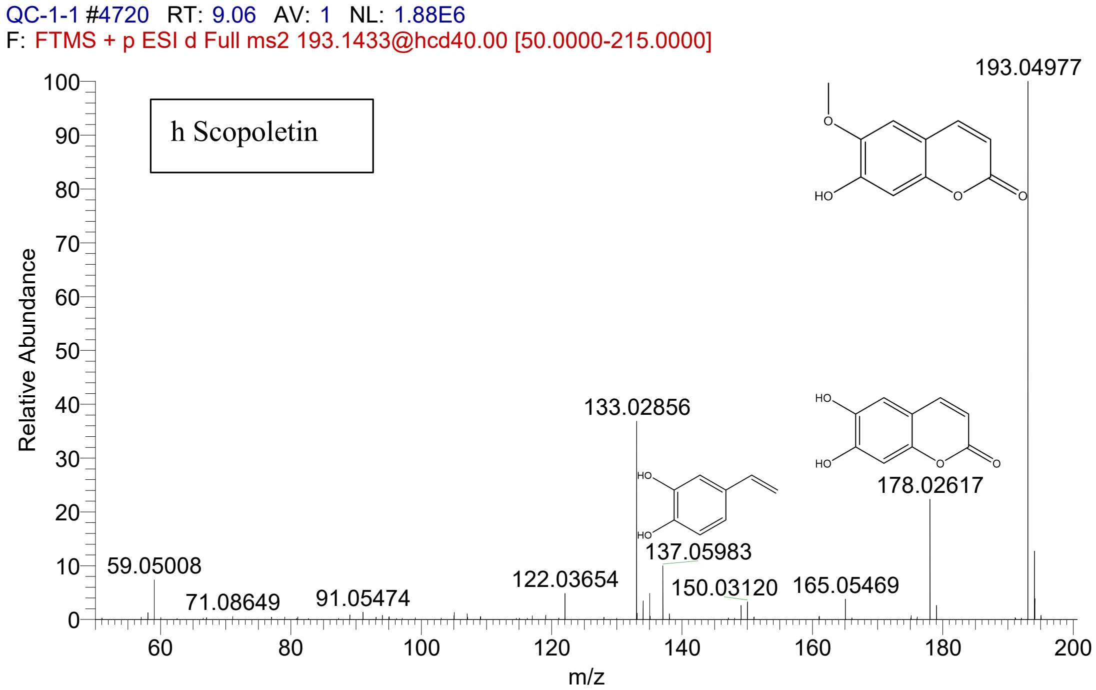
QC-1-1 #4720 RT: 9.06 AV: 1 NL: 1.88E6
F: FTMS + p ESI d Full ms2 193.1433@hcd40.00 [50.0000-215.0000]
h Scopoletin
193.04977
133.02856
178.02617
137.05983
122.03654
150.03120
165.05469
59.05008
71.08649
91.05474
Relative Abundance
m/z

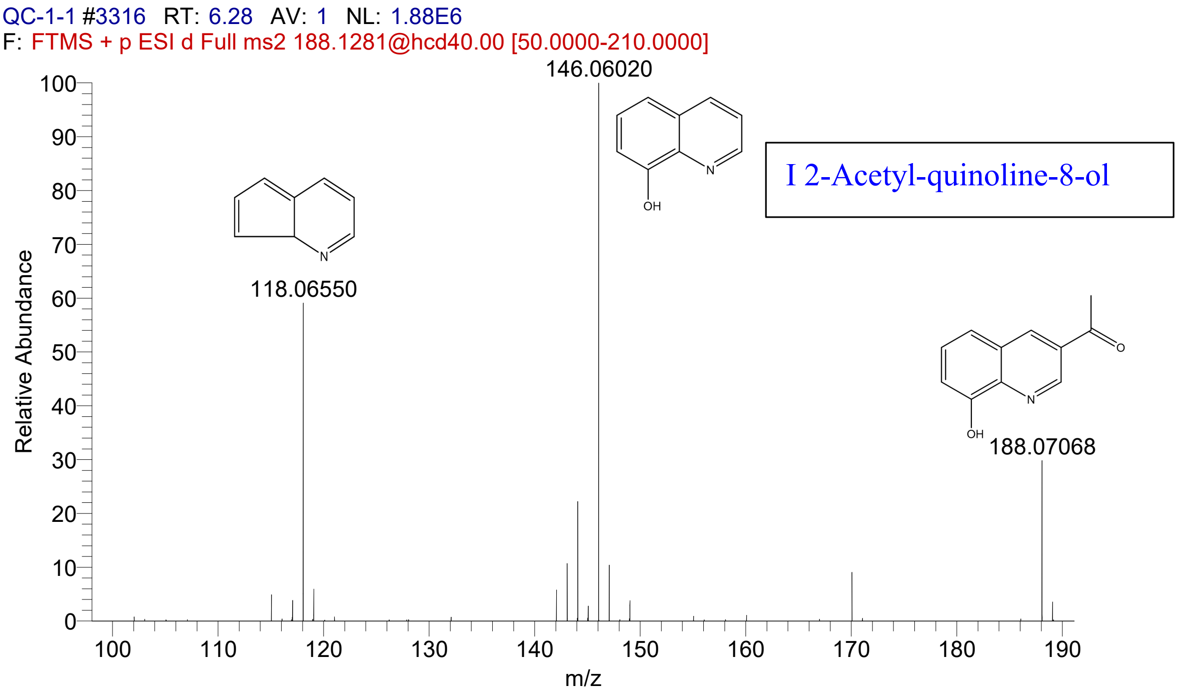

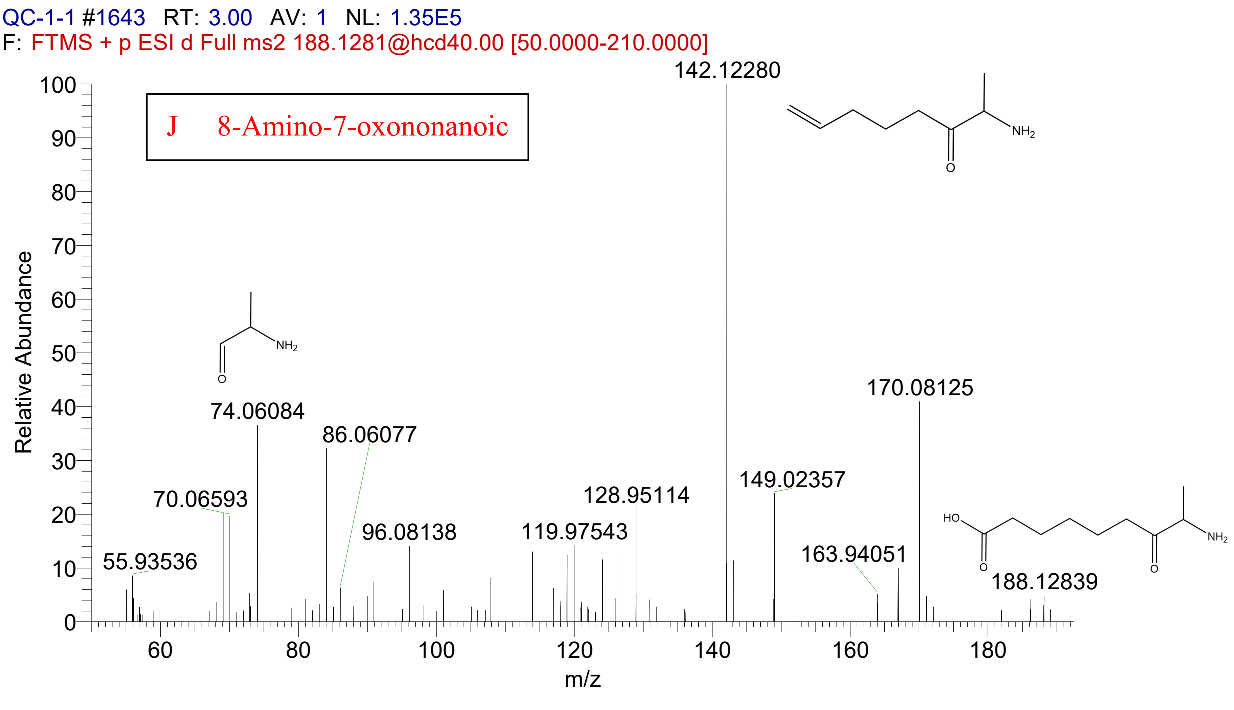

Figure S4 the scan mass spectrum and discriminant compounds of characteristic ion (b -g) in Honghe Dezhan hua.
