# Supplementary figures and images for: Traceability Research on Geographic Erigeron breviscapus Based on High-Resolution Mass Spectrometry and Chemometric Analysis
Source: Molecules. 2024 Jun 20;29(12):2930. doi: 10.3390/molecules29122930 (PMC11206744; doi:10.3390/molecules29122930)

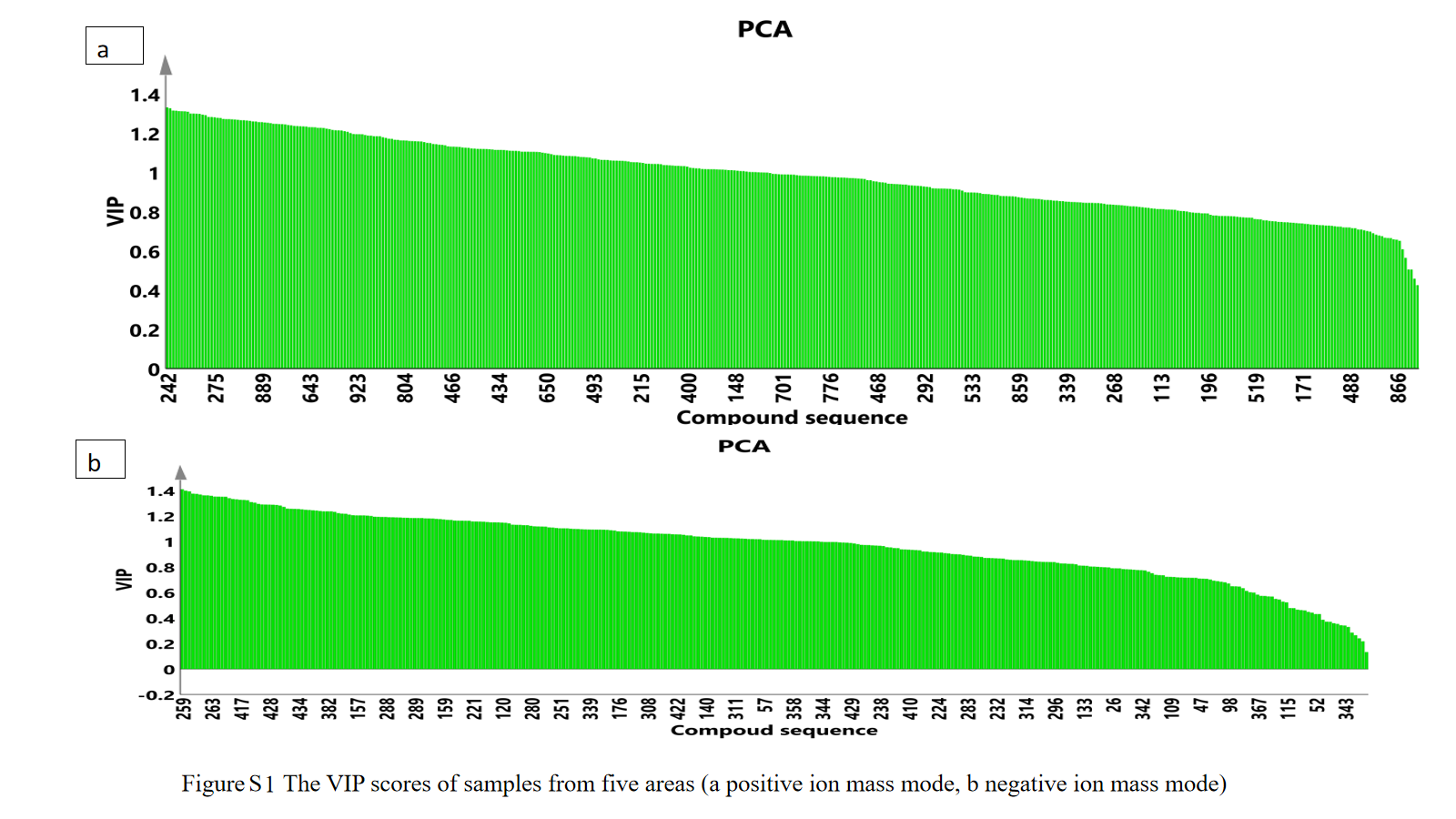

Supplement: Supplementary file 1 [file molecules-29-02930-s001.zip › Figure S1.png]

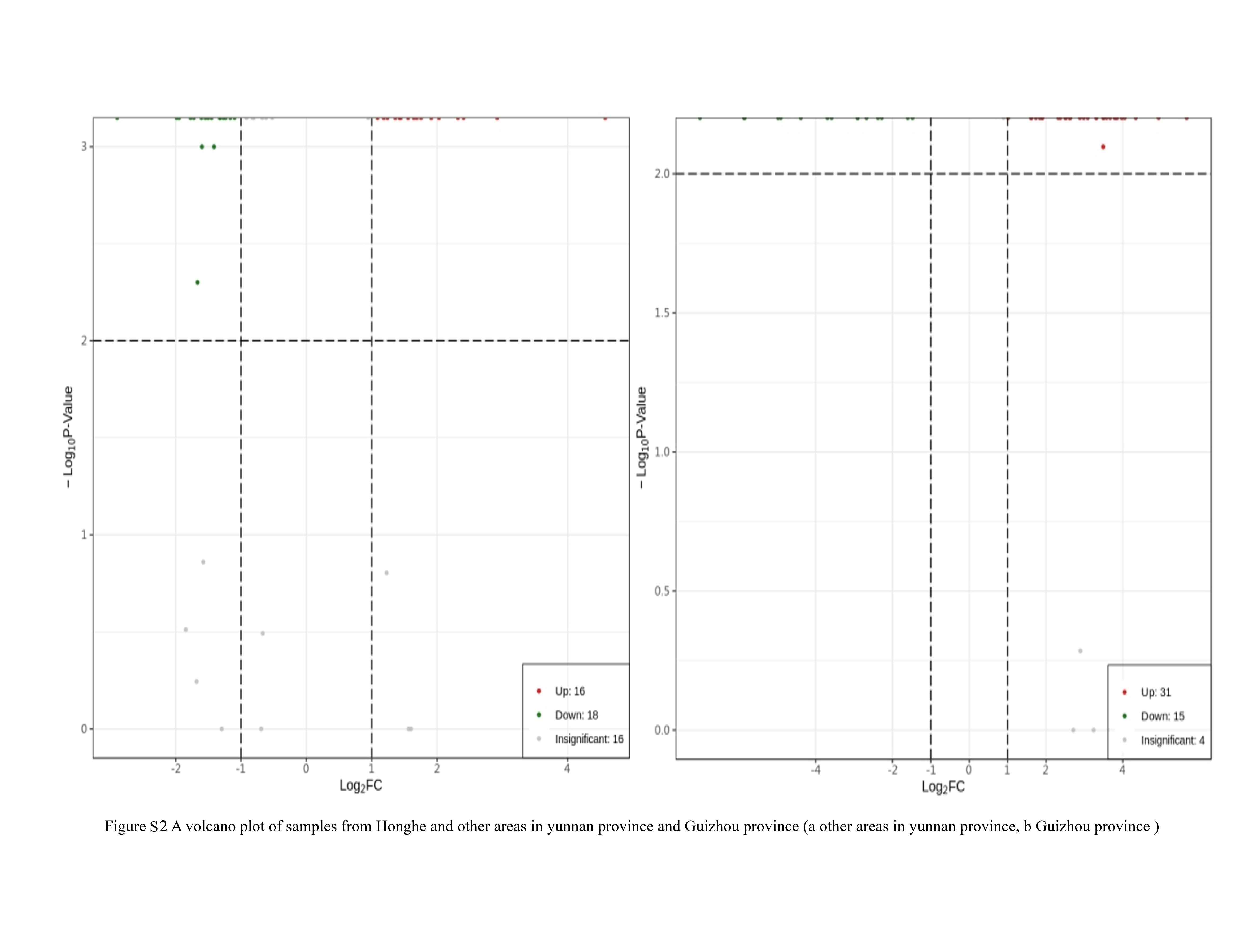

Supplement: Supplementary file 1 [file molecules-29-02930-s001.zip › Figure S2.png]

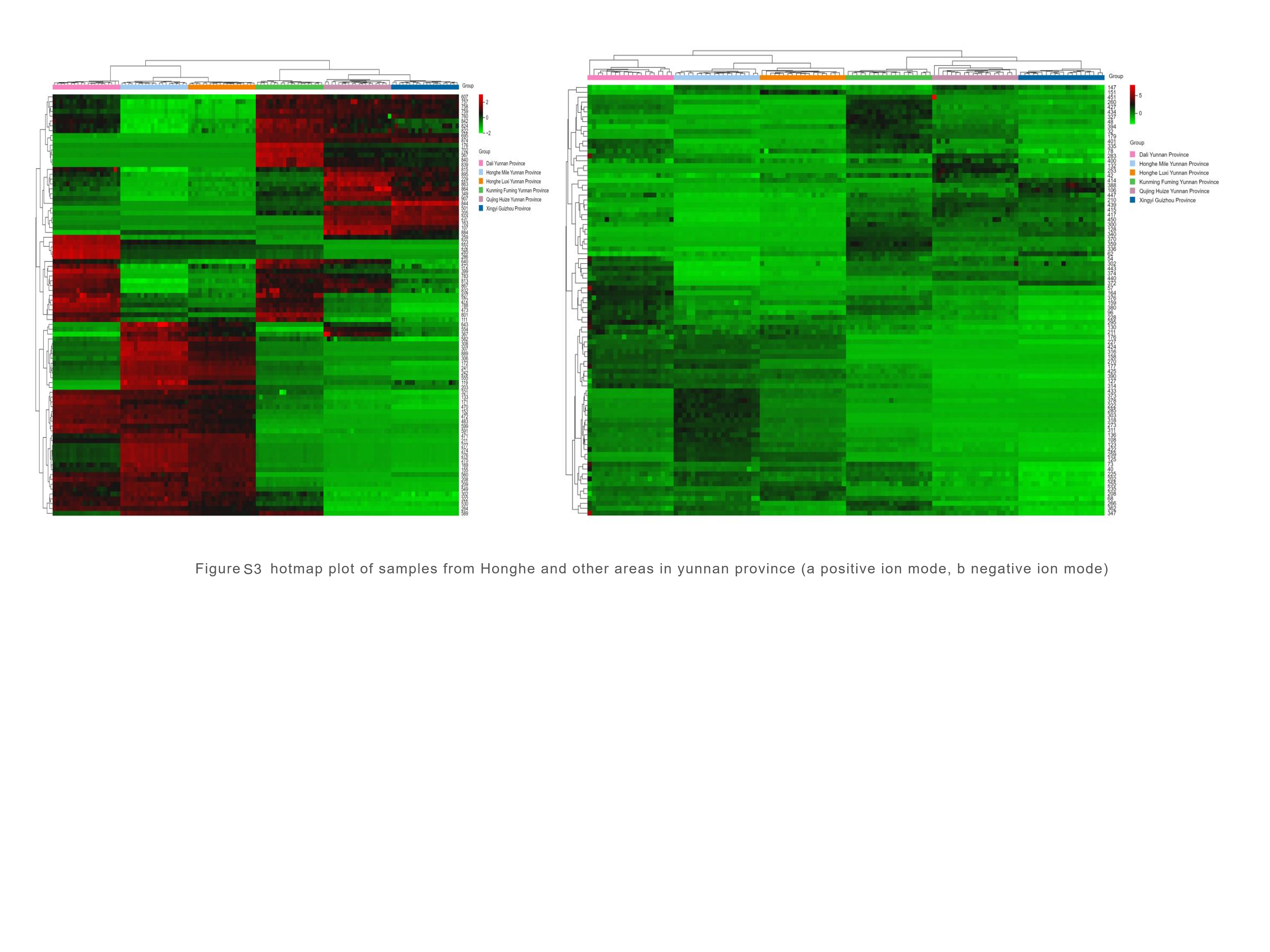

Supplement: Supplementary file 1 [file molecules-29-02930-s001.zip › Figure S3.png]
